# Predominance of interleukin-22 over interleukin-17 at the site of disease in human tuberculosis

**DOI:** 10.1016/j.tube.2011.06.009

**Published:** 2011-11

**Authors:** Kerryn Matthews, Katalin A. Wilkinson, Barbara Kalsdorf, Teri Roberts, Andreas Diacon, Gerhard Walzl, Janine Wolske, Mpiko Ntsekhe, Faisal Syed, James Russell, Bongani M. Mayosi, Rodney Dawson, Keertan Dheda, Robert J. Wilkinson, Willem A. Hanekom, Thomas J. Scriba

**Affiliations:** a Clinical Infectious Diseases Research Initiative, Institute of Infectious Diseases and Molecular Medicine, University of Cape Town, Observatory 7925, South Africa; b National Institute for Medical Research, Mill Hill, London, NW7 1AA, UK; c Clinical Infectious Diseases, Research Center Borstel, Borstel, Germany; d Molecular Biology and Human Genetics, Department of Biomedical Sciences, Stellenbosch University, Tygerberg 7505, South Africa; e Department of Medicine, Faculty of Health Sciences, University of Cape Town, Observatory 7925, South Africa; f University of Cape Town Lung Institute, Observatory 7925, South Africa; g Division of Medicine, Imperial College London, W2 1PG, UK; h South African Tuberculosis Vaccine Initiative and School of Child and Adolescent Health, Institute of Infectious Diseases and Molecular Medicine, University of Cape Town, Observatory 7925, South Africa

**Keywords:** Pleural tuberculosis, Pericardial tuberculosis, IL-17, IL-22, Inflammation

## Abstract

The inflammatory response to *Mycobacterium tuberculosis* (*M.tb)* at the site of disease is Th1 driven. Whether the Th17 cytokines, IL-17 and IL-22, contribute to this response in humans is unknown. We hypothesized that IL-17 and IL-22 contribute to the inflammatory response in pleural and pericardial disease sites of human tuberculosis (TB).

We studied pleural and pericardial effusions, established TB disease sites, from HIV-uninfected TB patients. Levels of soluble cytokines were measured by ELISA and MMP-9 by luminex. Bronchoalveolar lavage or pericardial mycobacteria-specific T cell cytokine expression was analyzed by intracellular cytokine staining.

IL-17 was not abundant in pleural or pericardial fluid. IL-17 expression by mycobacteria-specific disease site T cells was not detected in healthy, *M.tb-*infected persons, or patients with TB pericarditis. These data do not support a major role for IL-17 at established TB disease sites in humans.

IL-22 was readily detected in fluid from both disease sites. These IL-22 levels exceeded matching peripheral blood levels. Further, IL-22 levels in pericardial fluid correlated positively with MMP-9, an enzyme known to degrade the pulmonary extracellular matrix. We propose that our findings support a role for IL-22 in TB-induced pathology or the resulting repair process.

## 1 Introduction

Human infection with *Mycobacterium tuberculosis* (*M.tb*) is asymptomatic in most people and controlled, at least in part, by a T cell response comprising CD4 and CD8 cells, and expression of the Th1 cytokines, IFN-γ and TNF-α.^1^ However, this control fails in approximately 9.3 million people worldwide who develop clinical tuberculosis (TB) each year.^2^ Sites of established disease in humans represent compartments of failed immune control, offering the opportunity to study immune mediators and understand mechanisms of immune control in humans.

The inflammatory response is compartmentalized to the TB disease site, and is strongly Th1 driven. Significantly higher levels of IFN-γ, TNF-α and IL-12 are found in pleural fluid of TB pleuritis patients, compared with peripheral blood, or non-tuberculous effusions^3^ and antigen-specific Th1 cells are recruited to the pleural fluid.^4^ IFN-γ and TNF-α levels are also increased in another disease site, tuberculous pericardial fluid, compared with malignant and other infectious causes of pericarditis.^5^ Similarly, recruitment of IFN-γ expressing Th1 cells to the lungs of *M.tb*-infected individuals was observed after bronchoscopic instillation of *M.tb* purified protein derivative (PPD).^6^

Th17 cells, characterized by the expression of IL-17A, IL-17F and IL-22, are distinct from Th1 and Th2 cells. IL-17A was first described as a mediator of inflammatory diseases.^7^ This cytokine has since then been implicated in protective immunity against extracellular bacterial and fungal pathogens.^7^ IL-17 also plays a role in the control of murine *M.tb* infection as Th17 memory cells, induced by TB vaccination, mediated recruitment of protective Th1 cells to the lung by up-regulating chemokines.^8^ Upon infection, IL-17 may also trigger recruitment of neutrophils to the inflamed lung and facilitate granuloma formation.^9^ Notably, a recent study in cattle showed that induction of IL-17 responses after novel TB vaccination correlated with protection against *Mycobacterium bovis* TB.^10^

IL-22 is primarily expressed by CD4 T cells,^11^ although a variety of other immune cell subsets have also been shown to express this cytokine.^12,13^ IL-22 acts on epithelial cells and fibroblasts and may mediate deleterious or protective pro-inflammatory effects. While IL-22 has been shown to be a critical pro-inflammatory mediator of psoriasis,^14^ it also plays a role in host defense against extracellular bacteria, at mucosal surfaces of the lung and gut, as well as the skin.^15,16^ The role of IL-22 in immunity to mycobacteria is not well understood. A recent study in mice showed that IL-22 knock-out animals controlled *Mycobacterium avium* infection as well as wild type mice. Similarly, treatment of *M.tb* infected mice with neutralizing anti-IL-22 antibodies did not affect pathology, granuloma formation or bacterial burdens in the lung.^17^

However, recent studies in rhesus macaques reported upregulation of IL-22-expressing T cells in BAL and lungs upon *M.tb* infection^18^ and markedly increased IL-22 transcript expression in severe TB, compared with uninfected macaques.^19^ Further, IL-22-producing T cells were detected in lung tissue sections and granulomas of *M.tb* infected macaques.^18^ We recently reported that peripheral blood IL-17 or IL-22-expressing mycobacteria-specific CD4 T cells are induced in humans with *M.tb* infection or TB disease.^20^ We also observed increased IL-22 levels in BAL fluid from pulmonary TB patients, compared with healthy, *M.tb* infected controls. By contrast, IL-17 was undetectable by ELISA in BAL fluid from TB patients. Since BAL involves instillation of considerable volumes of saline, we reasoned that cytokines present at low levels may have been diluted to undetectable levels.^20^

We hypothesized therefore that IL-17 and IL-22 contribute to the inflammatory response at the disease site of human TB. We sampled undiluted fluid from two sites of TB disease, the pleura and the pericardium, in effusions from patients with pleural and pericardial TB, respectively. We also measured expression of these cytokines by BAL and pericardial cells.

## 2 Methods

### 2.1 Study participants and specimen collection

Patients were enrolled at 3 clinical sites in Cape Town, South Africa. The study was approved by the University of Cape Town Research Ethics Committee (References 402/2005 & 289/2007), the University of Stellenbosch Research Ethics Committee, and Lübeck. Written informed consent was provided by participants, all were HIV-1 seronegative.

Patients with pleural TB (*n* = 19, 11 M/8 F, mean age 39.4, range 20–59 years) were enrolled at the Division of Pulmonology of Tygerberg Academic Hospital, Cape Town. Pleural fluid, obtained via thoracentesis, and peripheral blood for separation of plasma were collected from all patients. TB was diagosed by a combination of clinical features and investigations including pleural fluid analysis (differential white cell count; ADA, LDH and protein content; cytological examination and culture), pleural biopsy with staining for acid-fast bacilli, culture and histopathological examination. Patients without a positive mycobacterial culture or without suggestive histopathology responded to TB treatment with a complete cure, strongly supporting the diagnosis.

Patients with pericardial TB (*n* = 22, 15 M/7 F, mean age 44.7, range 19–80 years) were recruited at the Cardiac Clinic in Groote Schuur Hospital, Cape Town. Pericardial TB was diagnosed by pericardial culture or PCR (positive for *M.tb*, *n* = 7) and/or clinical features, with symptoms and response to TB treatment (*n* = 15).^21^ These patients underwent pericardiocentesis with echocardiographic guidance for clinically indicated removal of their effusion. Pericardial fluid samples from additional patients without TB, who underwent open-heart surgery (*n* = 26, 7 M/19 F, mean age 51.8, range 23–80 years) were included as controls. Peripheral blood was collected from both groups for serum analysis.

Healthy adults (*n* = 8, 4 M/4 F, mean age 31.8, range, 21–42 years) recruited at the Khayelitsha Site B Clinic, Cape Town, underwent bronchoscopy to obtain bronchoalveolar lavage (BAL) samples as described previously.^22^ All subjects had evidence of latent *M.tb* infection, as measured by a positive peripheral blood mononuclear cell response to ESAT-6 and/or CFP-10 by IFN-γ ELISpot assay.^22^ Participants with any symptoms suggestive of TB, a history of TB or isoniazid preventive therapy, regular smoking, pregnancy, chronic cardiovascular or metabolic illnesses, immunosuppressive medication, and age below 21 years were excluded. All participants had negative cultures for *M. tuberculosis* in BAL and no radiological evidence of lung disease.

### 2.2 Analysis of soluble cytokines and MMP-9

Cells from pericardial or pleural fluids were removed by centrifugation and cell free fluids, plasma and serum samples stored at −80 °C. Soluble IL-17A (eBioscience), IL-22 (R&D Systems) and IFN-γ (BD Biosciences) were quantified by sandwich ELISA according to the manufacturers’ protocols. The lower detection limit for IL-17 was 7 pg/mL, for IL-22 was 15.6 pg/mL and for IFN-γ was 14 pg/mL. MMP-9 was quantified in cell-free pericardial fluid and serum by luminex analysis on the Bio-Plex platform (Bio-Rad Laboratories), using customized Fluorokine MultiAnalyte Profiling kits (R&D Systems), according to the manufacturer’s instructions.

### 2.3 BAL and pericardial cell analysis by intracellular cytokine staining

Processing and stimulation of fresh BAL mononuclear cells for intracellular cytokine staining was performed as previously described.^22^ *M.tb* PPD (SSI, Denmark), staphylococcal enterotoxin B (SEB, positive control, Sigma–Aldrich), and no antigen (negative control) were used. Pericardial cells were isolated from pericardial fluid by centrifugation, washed and resuspended in RPMI containing 10% fetal calf serum. These cells (2 × 10^6^ per condition) were stimulated for 18 h with the same antigens as described above for BAL cells. Brefeldin-A was added after 2 h. Cells were stained with fluorescent antibodies to CD3-Pacific Blue (clone UCTH1), CD4 PerCP-Cy5.5 (SK3) or CD4 Qdot 605 (SK3, Invitrogen), CD8 PerCP-Cy5.5 (SK1), IFN-γ-AlexaFluor700 (K3), IL-22 PE (142928, R&D Systems), all from BD Biosciences unless stated otherwise, and IL-17-AlexaFluor647 (eBio64CAP17, eBiosciences) at 4 °C for 60 min. Acquisition on a LSR II flow cytometer (BD Biosciences) and data analysis were performed as previously described.^22^

### 2.4 Data analysis

Statistical analyses were performed using Graphpad Prism software (version 5). Intergroup comparisons were performed using the Mann–Whitney *U* test, and paired data were analyzed using the Wilcoxon Signed Rank test. Associations were tested using the Spearman correlation test.

## 3 Results

### 3.1 IL-17 levels at the TB disease site

**Figure 1:**
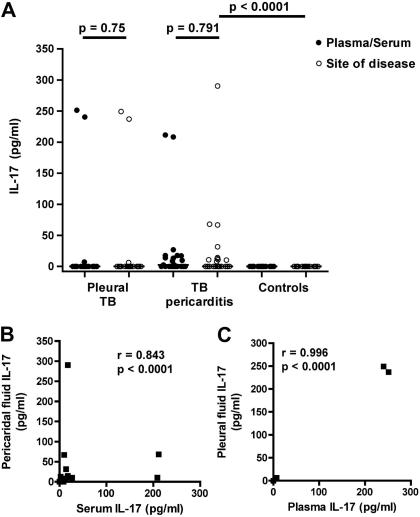
IL-17 at TB disease sites. (A) Levels of soluble IL-17 in pleural or pericardial fluid and matching plasma or serum levels from TB pleuritis patients or TB pericarditis patients, respectively. Differences were calculated using the Wilcoxon matched pairs test. Serum and pericardial fluid IL-17 levels from open heart surgery controls without TB are shown on the right (Controls). Differences between TB pericarditis and open heart surgery controls were calculated using the Mann–Whitney *U* test. (B and C) Associations between pericardial fluid and serum levels (B), or pleural fluid and plasma levels (C), of IL-17 from these patients. The Spearman test was used to test for correlations.

We quantified soluble IL-17 levels at established TB disease sites. IL-17 was undetectable in pleural effusions and in matching plasma samples from the majority of patients with pleural TB disease (Figure 1A). Only 2 patients had pleural fluid levels of IL-17 above the assay threshold (249 and 237 pg/mL, both *M.tb* culture negative). Notably, these 2 patients also had very similar levels of IL-17 in plasma (Figure 1A). The median IL-17 concentration in pericardial fluid from TB pericarditis patients was also 0 pg/mL (IQR 0-13.1, Figure 1A). However, 10 patients did have detectable pericardial IL-17 levels. IL-17 levels in matching serum samples were low, with a median of 2.5 pg/mL (IQR 0-17.3). Five of the patients with detectable pericardial IL-17 were *M.tb* culture positive, while the other 2 *M.tb* culture positive patients had no IL-17 in either serum or pericardial fluid. There was no significant association between *M.tb* culture positivity and detectable IL-17 at the site of disease (*p* = 0.172).

We also sampled pericardial fluid from patients without TB, as controls. IL-17 was undetectable in the pericardial fluid from 25 pericarditis controls, and their matching serum samples.

### 3.2 Lack of IL-17 compartmentalization to the disease site

No difference was found in IL-17 levels between disease site fluids and their matching plasma/serum samples, suggesting a lack of compartmentalization to the disease compartments (Figure 1A). Pericardial fluid and serum IL-17 levels correlated (Spearman *r* = 0.843, *p* < 0.001, Figure 1B). A positive correlation was also observed between pleural fluid and plasma levels (Spearman *r* = 0.996, *p* < 0.001), although this analysis was weakened by the undetectable levels of IL-17 in many patients. However, the latter analysis was not significant when the only 2 patients with detectable pleural fluid IL-17 were excluded (Figure 1C).

### 3.3 Elevated IL-22 levels at the TB disease site

**Figure 2:**
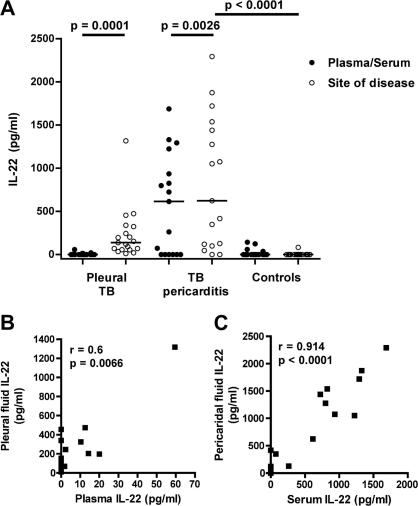
IL-22 at TB disease sites. (A) Levels of soluble IL-22 in pleural or pericardial fluid and matching plasma or serum levels from TB pleuritis patients or TB pericarditis patients, respectively. Serum and pericardial fluid IL-22 levels from open heart surgery controls without TB are shown on the right (Controls). (B and C) Associations between pericardial fluid and serum levels (B), or pleural fluid and plasma levels (C), of IL-22 from these patients.

We previously reported significantly higher IL-22 levels in BAL fluid from patients with pulmonary TB, compared with healthy controls.^20^ To investigate the role of IL-22 at other TB disease sites, we quantified soluble IL-22 in pleural and pericardial fluids from TB patients. Levels of IL-22 in pleural effusion and pericardial effusions from TB patients were readily detectable in most patients (Figure 2A). Only 1 pleural TB patient and 2 pericardial TB patients had undetectable effusion levels of IL-22. These fluid cytokine levels were higher than those in matched plasma and serum samples (Figure 2A). IL-22 levels were higher in pericardial effusions compared with pleural effusions (*p* = 0.04). We found no associations between IL-22 levels in pericardial fluid or serum and *M.tb* culture positivity (data not shown).

IL-22 was undetectable in the pericardial fluid from 24 of 25 control pericarditis patients without TB; 1 control patient had 84 pg/mL of IL-22 (Figure 2A).

IL-22 levels in pleural and pericardial fluids from TB patients correlated strongly with matching serum or plasma levels, respectively (Figure 2B and C).

Notably, we observed a positive correlation between pericardial fluid IL-22 and IL-17 levels (Table 1). Levels of these cytokines in pleural fluid did not correlate.

### 3.4 Elevated IFN-γ levels at the TB disease site

**Figure 3:**
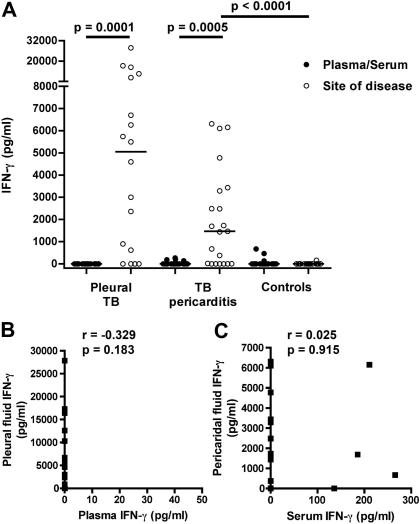
IFN-γ levels at TB disease sites. (A) IFN-γ levels in pleural or pericardial fluid and matching plasma or serum levels from TB pleuritis patients or TB pericarditis patients, respectively. Serum and pericardial fluid IFN-γ levels from open heart surgery controls without TB are shown on the right (Controls). (B and C) Associations between pericardial fluid and serum levels (B), or pleural fluid and plasma levels (C), of IFN-γ from these patients.

It is well established that the anti-mycobacterial response at the TB disease site is strongly Th1 driven.^3–6^ We detected high IFN-γ levels in pleural fluid samples from TB patients (median 5050 pg/mL, IQR 465-10,875) and in pericardial fluid from TB pericarditis patients (median 1450 pg/mL, IQR 0-3326) (Figure 3A). By contrast, no IFN-γ was detected in pericardial fluid from control pericarditis patients without TB. IFN-γ was not detected in plasma or serum from TB pleuritis and pericarditis patients, highlighting the compartmentalization of this Th1 cytokine to the inflammatory response at the TB disease site (Figure 3A). IFN-γ levels between disease site fluids and matching plasma or serum samples did not correlate (Figure 3B and C).

IFN-γ levels at either disease site were not associated with IL-22, or with IL-17 levels (Table 1).

### 3.5 IL-22 is associated with matrix metalloproteinase-9 levels

**Figure 4:**
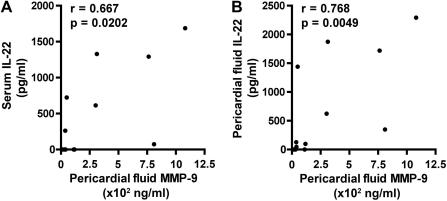
IL-22 levels correlate with MMP-9. (A) Serum IL-22 levels plotted against pericardial fluid MMP-9 levels from a subset of 12 TB pericarditis patients. (B) Pericardial fluid IL-22 levels plotted against pericardial fluid MMP-9 levels from these 12 TB pericarditis patients. The Spearman test was used to test for correlations.

Matrix metalloproteinase (MMP)-9 is a gelatinase, which was shown to degrade components of the extracellular matrix, resulting in tissue remodeling, destruction and/or pathology.^23^ Levels of MMP-9 in blood are associated with severity of TB disease; patients with extensive disease had the highest MMP-9 levels.^24^ To investigate whether IL-22 levels may be associated with mediators of lung pathology, we quantified MMP-9 levels in pericardial fluid and matching serum samples from 12 of the 22 TB pericarditis patients. Pericardial MMP-9 levels correlated positively with serum and pericardial levels of IL-22 from TB pericarditis patients (Figure 4).

### 3.6 IL-17-expressing CD4 T cells at disease sites

**Figure 5:**
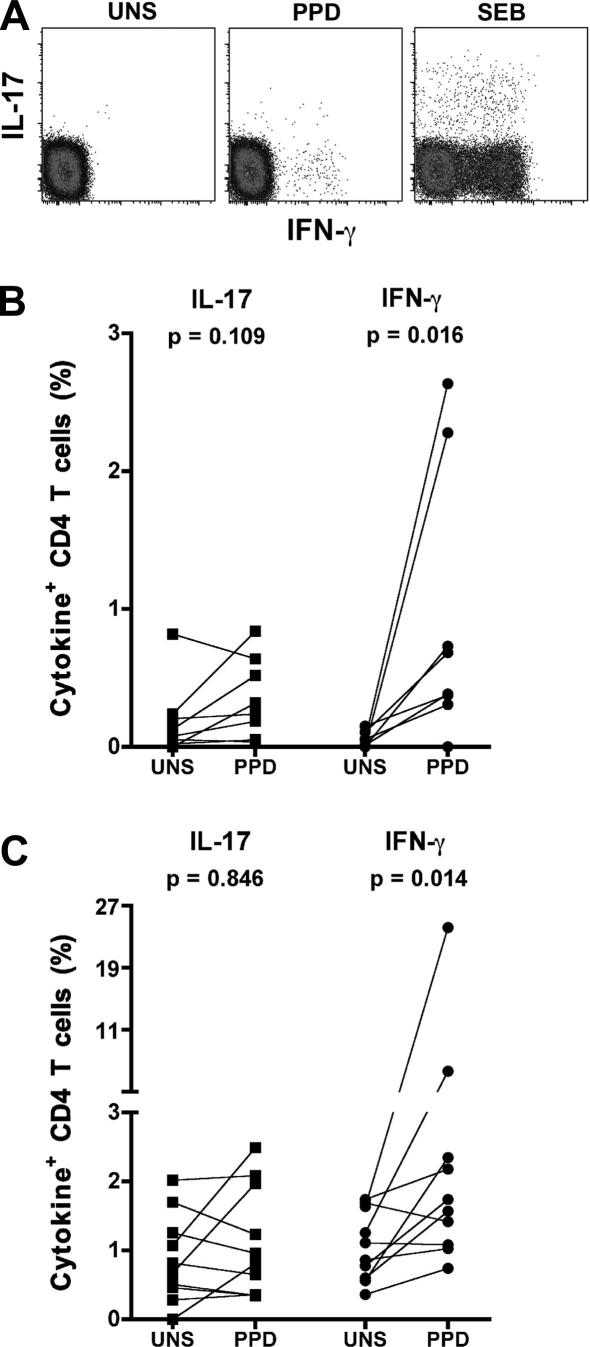
Flow cytometric detection of intracellular IL-17 or IFN-γ expression by BAL or pericardial CD4 T cells. BAL or pericardial cells were stimulated with medium, *M.tb* PPD or SEB and intracellular cytokine expression measured by flow cytometry. (A) Flow cytometry plots of BAL CD4 T cell expression of IL-17 and IFN-γ from a representative adult with *M. tuberculosis* infection. (B) Frequencies of IL-17 or IFN-γ expression by BAL CD4 T cells from 8 adults with *M. tuberculosis* infection. (C) Frequencies of IL-17 or IFN-γ expression by percardial CD4 T cells from 10 patients with TB pericarditis. Differences were calculated using the Wilcoxon matched pairs test.

Next, we used intracellular cytokine staining to detect mycobacteria-specific T cell responses at the disease site. IL-17 and IFN-γ expression were measured in BAL and pericardial cells after stimulation with PPD, SEB or no antigen (Figure 5A). Measurement of IL-22 expression was not reliable as no IL-22-expressing T cells were detected upon SEB stimulation (data not shown). These data were thus not included in the analysis. We detected no significant upregulation of IL-17 expression in PPD stimulated CD4 T cells relative to unstimulated cells from 8 healthy subjects with latent *M.tb* infection (Figure 5B). By contrast, frequencies of BAL CD4 T cells expressing IFN-γ upon PPD stimulation exceeded those in unstimulated cells. Similarly, frequencies of PPD stimulated IL-17-expressing pericardial CD4 T cells from 10 TB pericarditis patients were not different to unstimulated cells (Figure 5C). Frequencies of PPD-specific IFN-γ-expressing pericardial CD4 T cells again exceeded unstimulated frequencies.

## 4 Discussion

To investigate the role of the Th17 cytokines IL-17 and IL-22 in human TB, we quantified these cytokines in specimens from established sites of TB disease. Three major points emerged: (1) IL-17 was not abundant at sites of TB disease, and expression of IL-17 by PPD-specific CD4 T cells from disease sites was not detected, (2) IL-22 was readily detected in pleural and pericardial effusions at levels exceeding those detected in matching peripheral blood, and (3) levels of pericardial IL-22 correlated with MMP-9 levels in pericardial fluid and peripheral blood, suggesting a role for IL-22 in TB induced pathology.

Our data do not support a major role for IL-17 at established TB disease sites in humans. Firstly, we report that IL-17 is undetectable in pleural or pericardial effusions from most TB patients. These data corroborate our previous observation of undetectable IL-17 levels in BAL fluid from TB patients.^20^ Secondly, we found that IL-17-expressing BAL or pericardial CD4 T cell frequencies do not change upon PPD stimulation. By contrast, a significant increase in IFN-γ expression was detected in both BAL and pericardial CD4 T cells. These data suggest that mycobacteria-specific Th17 cells are infrequent or absent from disease sites. Thirdly, the lack of compartmentalization of IL-17 to disease sites argues against Th17 cell recruitment to the site of disease. Our results accord with the recent observation that IL-17 mRNA levels in alveolar lavage cells from TB patients and healthy controls are similar.^25^

These human data contrast with murine studies, which clearly report the presence of IL-17-expressing CD4 cells in lungs of *M. tuberculosis* infected mice.^8,9^ Upregulation of IL-17-expressing T cells in lung tissue was also reported upon *M.tb* infection in non-human primates.^18^ An important difference between our results and these animal studies may be the site of sampling. Fluid and cells from tuberculous pleural and pericardial effusions are not synonymous to granulomas. We also cannot exclude a possible role for IL-17 in immunological events that may occur early after *M.tb* infection as human studies invariably address established TB disease. Since murine studies are often based on short follow-up periods, the time between infection and sampling may account for some differences observed between mouse and human studies. Similarly, expression of IL-17 was measured up to 2 months after *M.tb* infection in the non-human primate study.^18^ A recent study in cattle also showed that IL-17 responses, induced by novel TB vaccination, correlated with protection against *M. bovis* TB.^10^ These authors measured IL-17 expression before and at 13 weeks after *M. bovis* infection. Longer follow-up in animal studies would shed more light onto whether IL-17 expressing T cells are maintained in established TB disease.

The low abundance of IL-17 in pleural or pericardial effusions may also result from IFN-γ-mediated suppression of IL-17 expression by Th17 cells.^20^ High levels of the Th1 cytokine, IFN-γ, are characteristic of the TB-associated inflammatory response.^4–6^ Our findings of high IFN-γ levels in fluids from disease sites reinforce this. However, we did not observe an inverse correlation between IFN-γ and IL-17 in those who had detectable IL-17 levels. Moreover, the correlation between pericardial fluid and serum IL-17 levels indicates an absence of disease site compartmentalization for this cytokine, unlike IFN-γ. Finally, it should be noted that immune responses observed in pericardial and pleural TB may be representative of more severe and/or different pathology than pulmonary TB.

Our finding of abundant IL-22 in pericardial and pleural fluids from TB disease sites raise the possibility that this cytokine may play an important role at the disease site of human TB. These data support our previous report of higher IL-22 levels in BAL fluid from TB patients, compared with low or undetectable levels in healthy controls.^20^ We observed higher IL-22 levels at disease sites compared with matching peripheral blood. These data are indicative of disease site compartmentalization, suggesting that IL-22-producing cells are either recruited to the disease site, or that IL-22 expression by resident cells is increased. In agreement with our finding, a recent study reported greater upregulation of IL-22-expressing T cells in BAL and lungs than in blood and lymphoid tissues upon *M.tb* infection of macaques.^18^ IL-22-producing T cells were also detected in lung tissue sections and granulomas of these *M.tb*-infected macaques. The lack of data on intracellular expression of IL-22 by disease site cells is a limitation of our study, as such data would have indicated whether CD4 T cells or other cell types are the source of IL-22 in humans.

Despite the reported increase of IL-22 protein and transcripts or IL-22-expressing cells in *M.tb* infection of macaques^18,19^ or in disease sites of humans with TB, it remains unknown what role IL-22 plays in host defense against *M.tb*. One possibility may be the well-described function of IL-22 as a regulator of keratinocyte mobility, epidermal differentiation and wound healing.^26–29^ The success of *M.tb* as pathogen is dependent on its destruction of the extracellular matrix. This process which is, at least partly, driven by MMPs brings about cavitation and thereby creates an immunoprivileged niche.^23^ We speculate that increased IL-22 at the TB disease site may be involved in the healing/regeneration response, rather than an anti-microbial immune mechanism, as was described for the extracellular bacterium, *Klebsiella pneumoniae*.^15^ Alternatively, the pro-inflammatory functions of IL-22 may mediate pathology; incubation of human colonic subepithelial myofibroblasts with IL-22 induced increased mRNA expression of several MMPs.^30^

The correlations of IL-22 in pericardial fluid with MMP-9 levels in pericardial fluid and blood support either hypothesis. Notably, peripheral blood MMP-9 levels were previously associated with severity of TB disease^24^ and higher levels of MMP-9 were detected in pleural fluid from patients with TB pleural effusions, compared with patients with congestive heart failure.^31^ Furthermore, MMP-9 was critical for granuloma formation in all stages of *Mycobacterium marinum* infection in zebrafish, and ESAT-6 interacted directly with epithelial cells to induce MMP-9^32^. Granulomas, originally thought to curtail infection by encasing mycobacteria, have also been shown to be a site for bacterial expansion.^33,34^

Additional investigation is required to understand the exact role of IL-22 in TB-associated lung pathology. Given that caseous necrosis and cavitation are not observed in *M.tb*-infected mouse lungs,^35^ such studies may best be done in animal models with TB pathology more similar to that seen in humans.

In summary, we present data that may not support a major role for IL-17 at established TB disease sites in humans, and propose that IL-22 may be involved in the TB-associated pathological or repair response.

## Competing interests

None.

## Authors’ Contributions

TJS, KAW, RJW and WH designed and coordinated the study. MN and BMM designed the clinical study of pericarditis. KM, KAW, RJW, WH and TJS wrote the manuscript. KM, TJS, JW and BK carried out the experiments and data analyses. TR, AD, GW, BK, JR, RD, KD, MN, FS, and BMM recruited participants and/or performed clinical procedures. All authors read and approved the final manuscript.

## Conflict of interest

None.

## Figures and Tables

**Table 1:** Associations between cytokine levels at each TB disease site. Significant correlations are in boldface.

	Pleural fluid		Pericardial fluid	
	IL-22	IFN-γ	IL-22	IFN-γ
IL-17	^∗^*r* = −0.231 (*p* = 0.342)	*r* = −0.172 (*p* = 0.495)	**r** **=** **0.559** (**p** **=** **0.020**)	*r* = 0.117 (*p* = 0.613)
IL-22	–	*r* = 0.324 (*p* = 0.190)	–	*r* = 0.130 (*p* = 0.632)

^∗^Spearman correlation analysis.
